# Labor induction with oxytocin in pregnant rats is not associated with oxidative stress in the fetal brain

**DOI:** 10.1038/s41598-022-07236-x

**Published:** 2022-02-24

**Authors:** Tusar Giri, Jia Jiang, Zhiqiang Xu, Ronald McCarthy, Carmen M. Halabi, Eric Tycksen, Alison G. Cahill, Sarah K. England, Arvind Palanisamy

**Affiliations:** 1 Department of Anesthesiology, Washington University School of Medicine, St. Louis, MO USA; 2 Department of Obstetrics and Gynecology, Washington University School of Medicine, St. Louis, MO 63110 USA; 3 Department of Pediatrics, Washington University School of Medicine, St. Louis, MO USA; 4 Department of Genetics, Washington University School of Medicine, St. Louis, MO USA; 5 Department of Women’s Health, Dell Medical School, The University of Texas at Austin, Austin, TX USA; 6 Present Address: Department of Anesthesiology, Beijing Chaoyang Hospital, Capital Medical University, Beijing, China; 7 Present Address: Department of Radiation Oncology, Washington University School of Medicine, St. Louis, MO 63110 USA

**Keywords:** Preclinical research, Translational research

## Abstract

Despite the widespread use of oxytocin for induction of labor, mechanistic insights into fetal/neonatal wellbeing are lacking because of the absence of an animal model that recapitulates modern obstetric practice. Here, we create and validate a hi-fidelity pregnant rat model that mirrors labor induction with oxytocin in laboring women. The model consists of an implantable preprogrammed microprocessor-controlled infusion pump that delivers a gradually escalating dose of intravenous oxytocin to induce birth at term gestation. We validated the model with molecular biological experiments on the uterine myometrium and telemetry-supported assessment of changes in intrauterine pressure. Finally, we applied this model to test the hypothesis that labor induction with oxytocin would be associated with oxidative stress in the newborn brain. Analysis of biomarkers of oxidative stress and changes in the expression of associated genes were no different between oxytocin-exposed and saline-treated pups, suggesting that oxytocin-induced labor was not associated with oxidative stress in the developing brain. Collectively, we provide a viable and realistic animal model for labor induction and augmentation with oxytocin that would enable new lines of investigation related to the impact of perinatal oxytocin exposure on the mother-infant dyad.

## Introduction

Oxytocin (Oxt) is one of the most widely used medications to induce labor and facilitate delivery. Between 1998 and 2007, the incidence of induction of labor more than doubled from 9.8 to 23%, with a large majority of patients receiving Oxt^1–3^. Taken together with a concomitant increase in the use of Oxt for augmentation of labor, approximately 50–70% of parturients are exposed to Oxt during labor^4,5^. Despite this widespread use for over 60 years, most research has focused on the contractile effects of Oxt on the uterus and associated obstetric outcomes^6–9^. Whether Oxt affects the fetus remains sparsely studied, despite evidence that it can reduce placental perfusion and enter the fetal circulation^10–17^.

An important scientific roadblock is the absence of an animal model that mirrors induction of labor in pregnant women with Oxt, one of the most common interventions in modern obstetric practice. This is in stark contrast to the availability of numerous animal models that recapitulate various facets of healthy and maladapted pregnancy. A major reason for the lack of an animal model for labor induction is the technical difficulty associated with delivering a gradually escalating dose of intravenous Oxt in a free-moving animal. Current preclinical studies examining the effect of Oxt on the fetus do so without inducing birth^16,18–21^, making them contextually less germane. Consequentially, the impact of pharmacological interventions during childbirth on the mother-infant dyad remains poorly investigated. In this report, we surmount these challenges and present a hi-fidelity pregnant rat model for elective labor induction and augmentation with Oxt using an implantable, programmable, microprocessor-controlled precision drug delivery pump to closely mimic obstetric management of human labor. We chose a rat model because of (i) anatomical similarities between the rat and the human placenta (hemochorial)^22,23^, (ii) better characterization of Oxt metabolism in the rat uterus^24^, (iii) a relatively larger size which makes multiple invasive procedures (pump implantation, internal jugular vein catheterization) less challenging, and (iv) the possibility of leveraging the rich social behavioral repertoire of rats for future neurobehavioral studies in the offspring^25,26^.

Recent epidemiological studies suggest a modest but controversial association between labor induction and neurodevelopmental disorders such as autism spectrum and attention deficit hyperactivity disorders in the offspring^27–33^. An outstanding concern with these studies is the inability to distinguish the need for labor induction from its possible effects, and to delineate the effect of an individual pharmacological agent from a combination of drugs used for labor induction. With the rising popularity of elective induction of labor^9^, where labor is not induced for maternal or fetal indications, there is a compelling need to simultaneously pursue preclinical investigations to better understand the impact of these practices on long-term neurodevelopmental health of children. Here, we endeavor to address this knowledge gap by performing foundational experiments regarding the potential mechanisms by which labor induction with oxytocin can impact the developing brain. We rule out oxidative stress as a likely possibility, suggesting that the effects of Oxt on the fetus, if any, are likely to be directly mediated.

## Results

### Development of the model for labor induction with Oxt

**Figure 1:**
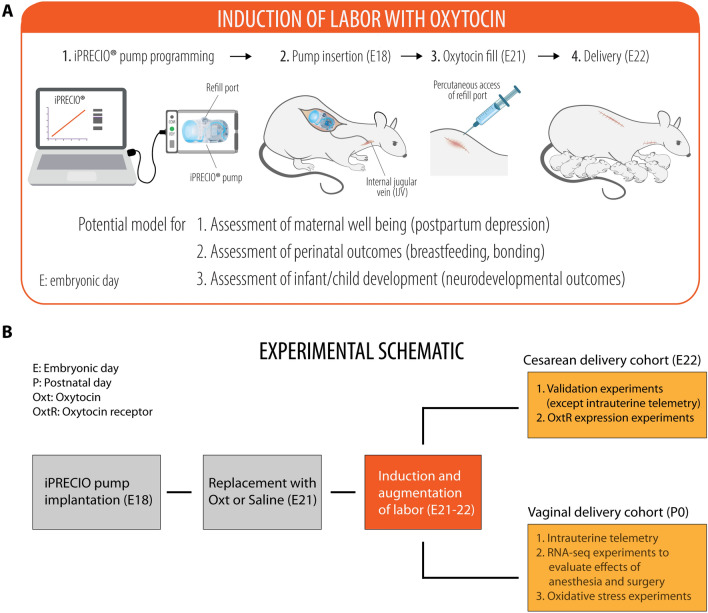
Experimental schematic for labor induction with oxytocin in term pregnant rats. (**A**) A cartoon depicting the programming and implantation of iPRECIO pump in a pregnant rat followed by birth of healthy pups. (**B**) Experimental schematic showing the overall experimental outline with two separate cohorts for cesarean and vaginal delivery. In the vaginal delivery cohort, there were three sets of independent experiments for (i) intrauterine telemetry, (ii) RNA-seq experiments to assess the impact of in utero exposure to anesthesia and surgery on the cortical transcriptome of the newborn brain, and (iii) examination of oxidative stress in newborn pups after either Oxt or saline, respectively.

**Figure 2:**
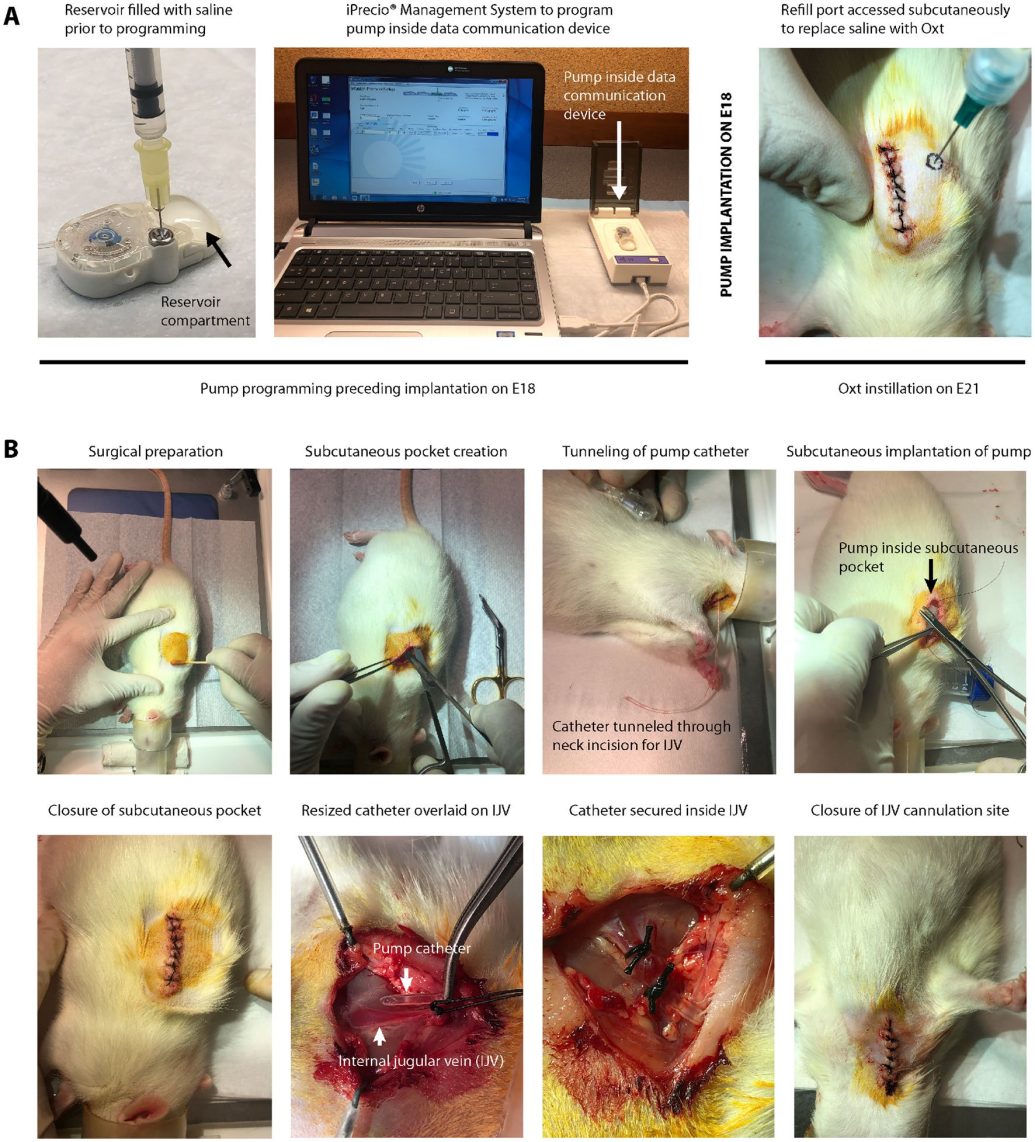
Workflow of experimental and surgical procedures associated with creation of the model. (**A**) Workflow for iPRECIO pump programming prior to implantation on E18 and subsequent replacement of saline with Oxt by subcutaneous access of the refill port on E21. (**B**) Left to right: sequential surgical workflow for implantation of the pre-programmed iPRECIO pump followed by internal jugular vein cannulation with the pump catheter on E18. All surgical procedures were performed in strict accordance with institutional guidelines for rodent surgery, anesthesia, and analgesia.

Overall, 44 timed-pregnant Sprague Dawley dams were used for the study (Supplementary Table S1). The experimental schematic is provided in Fig. 1. A detailed step-by-step description of the surgical procedure is provided along with a photo montage in Fig. 2. A video walkthrough of the experimental setup is presented as Supplementary Movie S1. With the final regimen for Oxt as described in Methods, dams gave birth to pups predictably within 8–12 h. Litter size and weight gain trajectory of the offspring from one experimental cohort are presented in Supplementary Table S2. Handling of critical steps and troubleshooting are described in greater detail in the Supplementary Materials and Methods.

### Validation of the model

**Figure 3:**
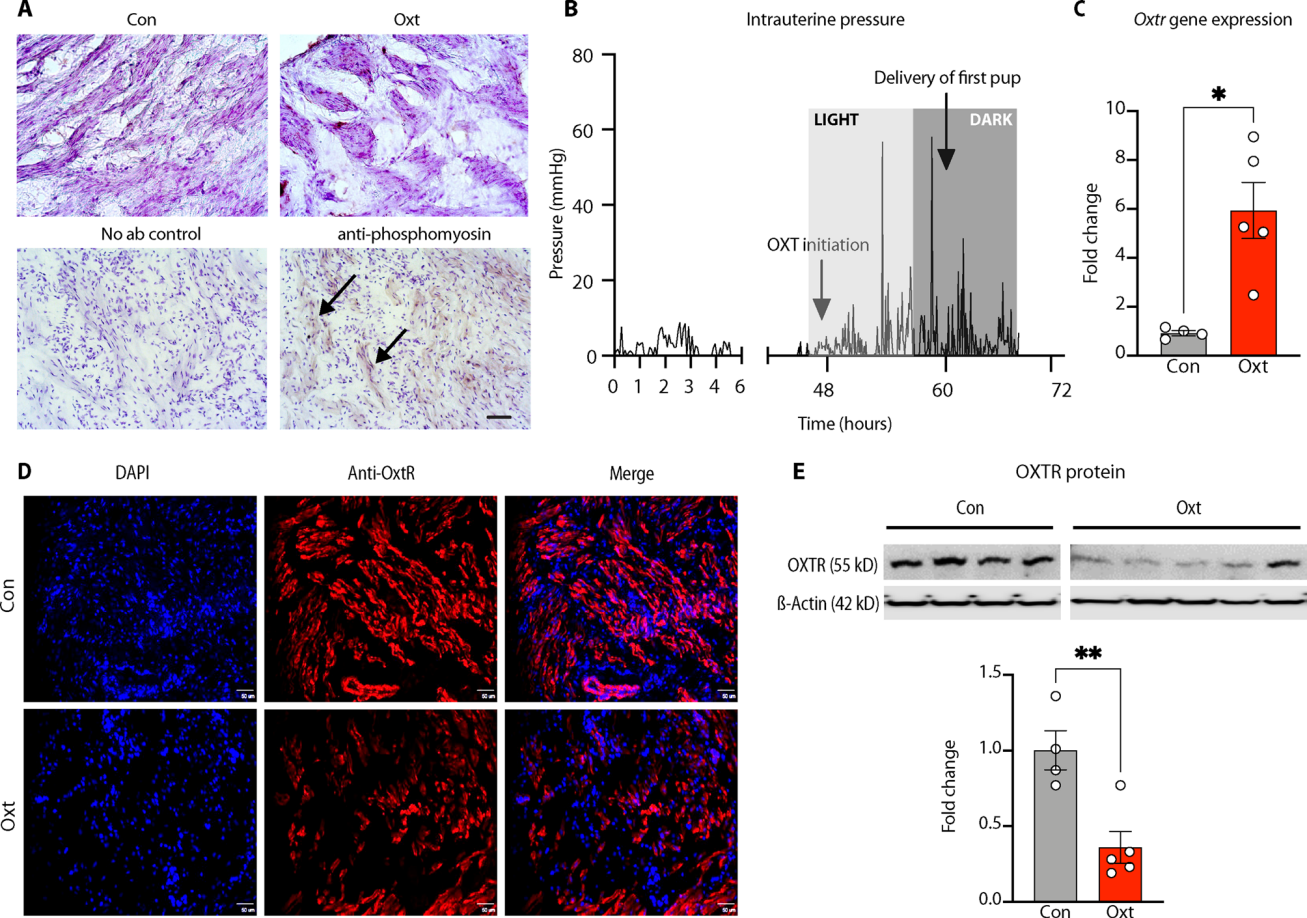
Validation of the model for labor induction with oxytocin. (**A**) Visualization of uterine contraction. Upper panel: uterine myometrium harvested from pregnant E21 rats at least 8 h after either saline (control) or intravenous Oxt infusion and stained with hematoxylin. 20 × photomicrographs showing lack of clustering of uterine myocytes in saline-treated myometrium (left) compared to extensive clustering in the Oxt-exposed myometrium (right). Lower panel: 5-μm frozen sections from Oxt-exposed myometrium stained without (left) or with rabbit anti-mouse phosphomyosin light chain kinase showing prominent staining among clustered uterine myocytes revealed with anti-rabbit HRP conjugate (marked by arrows) (right). Nuclei counterstained with hematoxylin. Scale bar = 100 μM. (**B**) Labor induction with Oxt causes cyclical increases in intrauterine pressure. Labor was induced with Oxt at 48 h after pump implantation (around 12 noon, light cycle, E20) and intrauterine pressure changes were monitored with telemetry. Oxt initiation was associated with acute and cyclical increases in intrauterine pressure until birth (first pup delivered around 21:00 h, dark cycle, E20). Light and dark cycle from 07:00–19:00 and 19:00–07:00, respectively. (**C**) Labor induction with Oxt was associated with a significant increase in OxtR gene expression at 8–12 h. (**D**) Labor induction with Oxt decreases OxtR immunoreactivity in the rat uterus. Sample 20 × photomicrographs from 5-µm sections of the uterine myometrium stained with goat anti-rat OxtR antibody (1:100) and revealed with Alexa Fluor® 594 labeled rabbit anti-goat antibody (1:300). Note naïve uterine myometrium with bright staining for OxtR in the upper panel, in sharp contrast to Oxt-exposed myometrial tissue in the lower panel where staining was scant, suggesting downregulation of OxtR. Scale bar = 50 μM. (**E**) Representative cropped western blot images showing a decrease in membrane associated OxtR protein expression after labor induction with Oxt and quantified with densitometry. Full length blot is presented in Supplementary Figure S3. Please note that the Oxt and Con lanes have been cropped and reversed to present the data in a manner consistent with the rest of the manuscript (i.e., Con followed by Oxt data). Data analyzed with Welch’s t-test and presented as mean ± SEM; *p ≤ 0.05, **p ≤ 0.01.

The best validation of our model was the successful vaginal delivery of thriving pups within 12 h after initiation of the Oxt regimen (Supplementary Movie S2). In addition, we confirmed the presence of immunoreactive phosphorylated myosin light chain kinase (MLCK)^34^, a serine/threonine kinase and a downstream regulator of the effects of Oxt on the actin-myosin ATPase, in Oxt-exposed myometrium (Fig. 3A). Next, we confirmed that Oxt initiation was accompanied by a rise in intrauterine pressure, a *sine qua non* feature of labor^35–37^, and lasting until birth of all pups (Fig. 3B). This was associated with an increase in OxtR gene expression in the uterine myometrium (Fig. 3C). In contrast, exposure to Oxt for at least 8 h resulted in a decrease in OxtR immunoreactivity (Fig. 3D) and membrane bound OxtR protein expression (Fig. 3E) similar to human data. Collectively, we established the translational relevance of our model by mirroring both Oxt management of labor and its effect on the uterine myometrium.

### Effect of surgery and anesthesia during pump implantation on the developing brain

**Figure 4:**
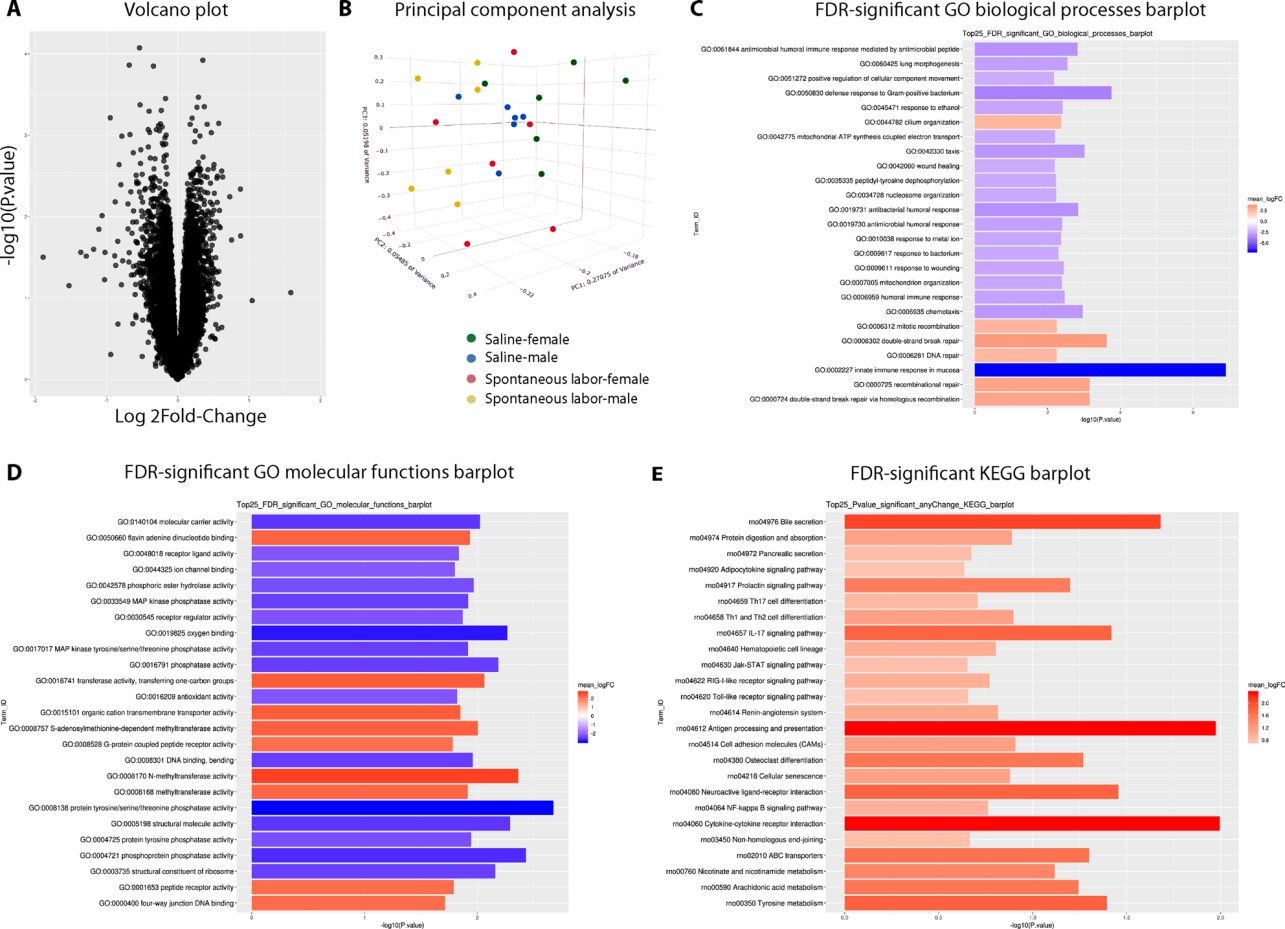
Impact of anesthesia and surgery on the newborn cortical transcriptome. (**A**) Volcano plot showing the absence of significantly differentially expressed genes between the spontaneous labor vs. saline pump groups (n = 6/treatment condition from 3 litters each). (**B**) Principal component analysis (PCA) showing that the major source of variance is not the treatment condition but the sex of the offspring, albeit not significant (green: saline/female, blue: saline/male, red: spontaneous labor/female, yellow: spontaneous labor/male). (**C**, **D**) Top 25 false discovery rate-adjusted significantly up- and downregulated genes for Gene Ontology (GO) biological processes (C) and molecular functions (D) after labor induction with Oxt. (**E**) Significantly upregulated genes with Kyoto Encyclopedia of Genes and Genomes (KEGG) analysis. GO and KEGG analyses revealed a differential impact of anesthesia and surgery on multiple pathways, mostly related to oxygen binding and the immune response, respectively. Therefore, for the rest of our experiments, we used saline pump-implanted dams that eventually labored spontaneously as controls, instead of dams that labored spontaneously without exposure to anesthesia and surgery. The mean log twofold-change in the GO and KEGG barplots are the mean log twofold-change of the genes within the term versus the mean log twofold-changes of the genes in the background using the GAGE method of testing log twofold-changes for perturbations in expression.

Because exposure to anesthesia and surgery can affect the developing brain^38–42^, we compared the cortical transcriptomes of newborn pups born to spontaneously laboring dams that were not exposed to pump implantation surgery *vs*. those that were implanted with a saline-filled pump on E18 (and therefore requiring anesthesia). Unbiased RNA-seq analyses of the cerebral cortex of vaginally delivered newborn pups revealed no significant changes in the cortical transcriptome after exposure to surgery and anesthesia as shown by the lack of significantly differentially expressed genes in the volcano plot (Fig. 4A; heat map in Supplementary Fig. S1). Principal component analysis (Fig. 4B) revealed that the major source of variance was not the treatment condition but the sex of the offspring, albeit not significant. Top up- and downregulated genes from GO and KEGG analyses are presented in Fig. 4C–E. A comprehensive list of differentially expressed genes and unadjusted p-value significant differentially expressed genes is provided in Supplementary Data S1 and S2, respectively.

### Examination of the redox state of the fetal cortex after labor induction with Oxt

**Figure 5:**
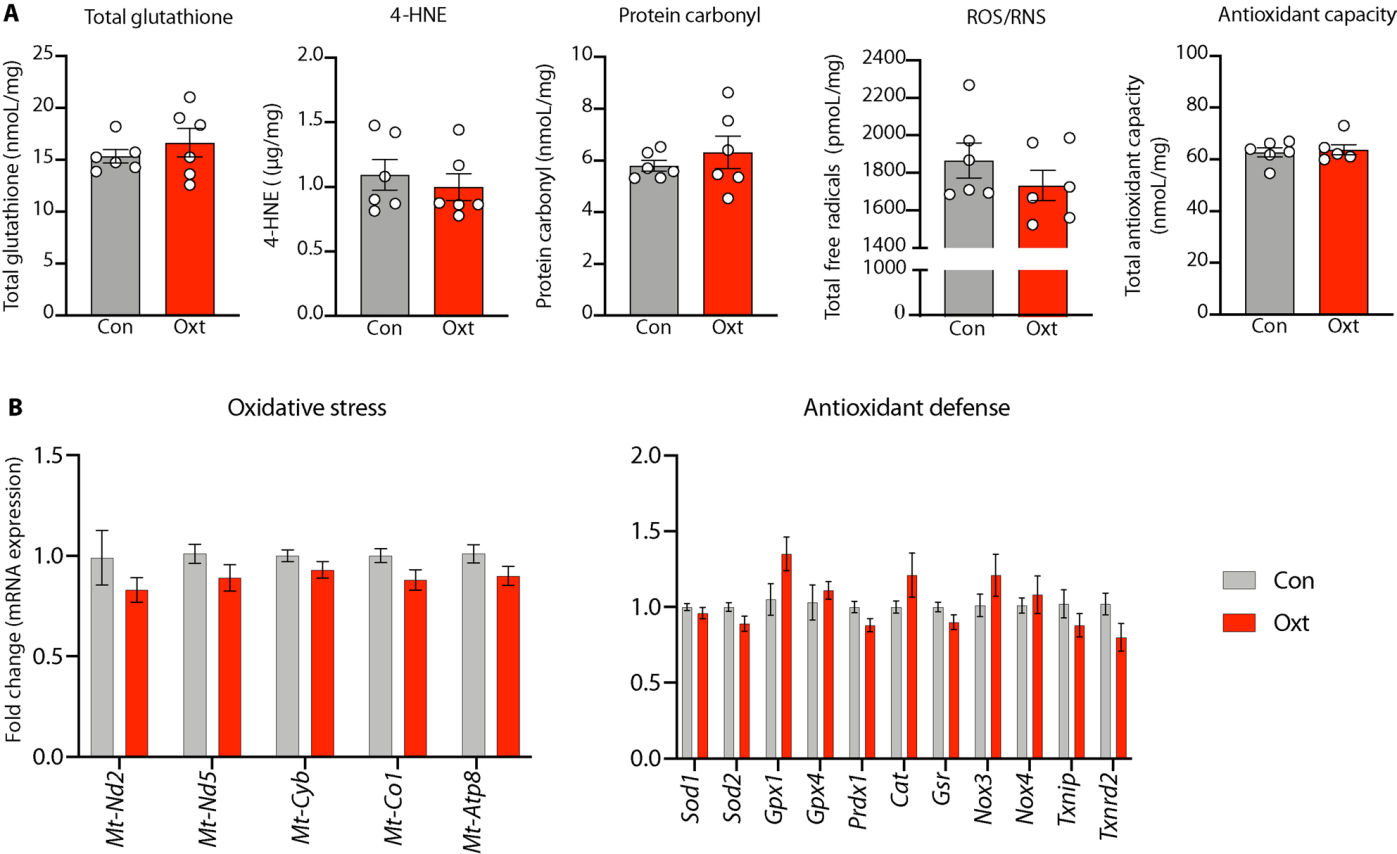
Labor induction with oxytocin is not associated with oxidative stress in the developing brain. (**A**) From left to right: Labor induction with oxytocin was not associated with an increase in either glutathione, 4-hydroxynonenal, protein carbonyl, reactive oxygen/nitrogen species (ROS/RNS), or total antioxidant capacity in the developing fetal cortex. All data are presented per mg of brain protein. Fetal brains exposed to paraquat in utero served as positive control. (**B**) Expression of genes mediating oxidative stress or antioxidant defense were not significantly differentially expressed in the fetal cortex after labor induction with oxytocin. Collectively, these data indicate that labor induction with Oxt is unlikely to be associated with oxidative stress in the developing brain. Data were analyzed with Welch’s t-test and expressed as mean ± SEM (n = 5–8 per treatment condition with pups represented from all unique dams).

Labor induction with Oxt was not associated with changes in the concentration of total free radicals, 4-hydroxynonenal or protein carbonyl, in the newborn cortex. Nor were there any significant differences in antioxidant capacity; both glutathione and total antioxidant capacity were unchanged after Oxt (Fig. 5A). Furthermore, we did not observe any significant changes in the expression of emblematic genes pertinent to the oxidative stress/antioxidant pathway (Fig. 5B; TaqMan qPCR probe list in Supplementary Table S3). Collectively, these data provide reassurance that the use of Oxt for labor induction is unlikely to be associated with oxidative stress in the fetal brain.

## Discussion

In this report, we present a realistic and tractable animal model for labor induction with Oxt. In addition to functional validation of the model, we were able to demonstrate features consistent with the use of Oxt in human labor: (i) a decrease in OxtR protein expression in the uterine myometrium, and (ii) confirmation of increased intrauterine pressure with Oxt. Furthermore, we provide evidence for the translational utility of the model by showing that labor induction with Oxt was not associated with oxidative stress in the fetal brain.

Ethical and logistic challenges significantly limit the scope of mechanistic research on pregnant women and their newborn. Our contextually relevant animal model, by providing unrivaled access to maternal and fetal tissue, has wide-ranging implications for translational research related to perinatal Oxt exposure. In the absence of a clinically relevant animal model for labor induction with Oxt, our work provides the opportunity to investigate lingering concerns about the impact of Oxt on neurobehavioral development of the offspring^27,33,43,44^, epigenetic regulation of OxtR in the fetal brain^16^, relationship between intrapartum Oxt use and breastfeeding success^45–47^, and the complex association between Oxt and postpartum depression^48–50^. Furthermore, by scaling down with appropriate equipment (iPRECIO SMP 310-R with a dedicated wireless communication device), transgenic mouse models could be used to investigate complex gene-environment interaction studies in the perinatal period. Motivated by recently published work in on the fetal effects of Oxt in prairie voles and mice^16,21^, ongoing studies in our laboratory are focused on the transfer of maternally administered Oxt across the placental and fetal blood–brain barriers and its impact on Oxt-ergic signaling in the fetal brain.

The biggest strength of our model is how it mirrors labor induction with Oxt in clinical practice. Though we prefer not to anthropomorphize our study (because biological validation of the effect of Oxt via birth of living pups was our endpoint), we provide information on the cumulative Oxt dose for reassurance that it was not abnormally high by orders-of-magnitude. Approximately 3–7 μg Oxt was required to induce birth, which is comparable to the dose ranges typically used during human labor. For example, parturients receive on average, a cumulative Oxt dose of 2000–4000 mIU or 2–4 IU (IU = International Unit) during the course of labor^51^. Because 1 IU = 1.68 μg of Oxt peptide, this would translate to approximately 3.4–6.8 μg of Oxt, similar to what we used in our model. Nevertheless, considering that we took a ‘top-down’ approach and settled for the first Oxt infusion regimen that was associated with birth of healthy pups without labor dystocia, it is likely that the cumulative Oxt dose is relatively higher. Despite this, our model simulates clinical practice to a large extent, and it is likely that research knowledge generated using this model is more likely to provide reliable and actionable mechanistic data than other currently available models.

As a G protein-coupled receptor that is sensitive to downregulation, our findings of reduced membrane bound OxtR protein expression after Oxt exposure is broadly consistent with published data in human studies^6^. However, to our surprise, expression of the OxtR gene was significantly increased after labor induction with Oxt. We believe that these apparently contradictory findings could be due to the choice of myometrial samples by Phaneuf et al.^6^; samples were collected from patients who underwent cesarean delivery after dystocic labor with Oxt suggesting the possibility of abnormal transcription during intrapartum arrest of labor. In contrast, we performed cesarean delivery during uncomplicated labor to facilitate sample collection. This line of thought is supported by the 4-fivefold increased myometrial expression of OxtR gene during uncomplicated labor in rodents^52,53^. The only other relevant preclinical study that examines this question is that of Hirayama et al. which used an osmotic pump to deliver a continuous subcutaneous infusion of Oxt in pregnant mice^54^. However, the experimental paradigm did not allow for escalation of Oxt dose, nor did the study assess the impact of Oxt administration on the uterine myometrium. Furthermore, the pup survival rate was low, suggesting the possibility that the high dose of Oxt could have caused tetanic, rather than cyclical, uterine contraction, labor dystocia, and fetal demise. That this is a strong possibility is reinforced by similar observations during the iterative phase of model creation in our study; higher doses of Oxt were associated with significant dystocia and perinatal demise of the pups. Our demonstration of cyclical changes in intrauterine pressure after initiation of Oxt, predictable delivery of alive and thriving pups, and a nurturing mother, provides reassurance that our model most closely resembles pharmacological management of human labor.

Epidemiological evidence, albeit conflicting, suggests a link between the use of Oxt and neurodevelopmental disorders such as autism spectrum disorder (ASD)^27,28,32,33^. Though the mechanisms remain unexplored, both oxidative stress and a direct effect of Oxt on the Oxt receptor in the fetal brain have been suggested as possibilities^33,43,44^. We focused on oxidative stress because Oxt causes a higher rate of rise in intrauterine pressure than spontaneous labor^11,36^, and is reported to cause lipid peroxidative injury^55^, decrease the anti-oxidant glutathione in cord blood^56^, and increase amniotic fluid lactate^57,58^. Because the developing brain is vulnerable to oxidative stress^59^, we applied our model to determine if induction and augmentation of labor with Oxt was associated with oxidative stress. Our findings were reassuring in that even after 8–12 h of exposure to Oxt-induced uterine contractions, there was no evidence for oxidative stress in the newborn brain. Lack of oxidative stress after prolonged exposure to repetitive Oxt-induced uterine contractions in a polytocous species where labor typically lasts between 90 and 120 min^60^, gives us more confidence that this is unlikely to be a concern for the human fetus. Taken together, our preclinical data suggest that the impact of Oxt on the fetus, if any, is likely to be a direct effect via changes in Oxt-ergic signaling rather than an indirect effect via oxidative stress. This notion is partly supported by evidence for changes in DNA methylation of the OxtR and downregulation of the OxtR gene in fetal brains exposed to Oxt in utero^16,21^.

Our research has a few limitations. First, our model can be perceived as contrived. Considering the technical challenges of delivering an escalating dose of intravenous Oxt in a free-moving animal to simulate obstetric practice, we considered all possibilities before pursuing this model. Importantly, our model is in no way more traumatic or less realistic than the unilateral carotid artery ligation/ anoxia model to investigate perinatal asphyxia in rodents^61,62^. Second, is the poor neurodevelopmental correlation between rats (altricial) and humans (semi-precocial) which would limit some of the translational aspects of our work. This could be partially offset by performing Oxt exposure studies in postnatal pups at day 10 or beyond, but it would, however, be limited by the lack of context and the absence of Oxt-provoked strong uterine contractions that precede delivery. More importantly, viewing all events solely from a neurodevelopmental perspective ignores the vastly more important evolutionary role of oxytocin during the birthing process as a facilitator of intrauterine to extrauterine transition of life (adaptation to birth hypoxia, lactation, thermoregulation etc.)^63,64^. Nevertheless, we expect our work to inspire other researchers to either develop a more neurodevelopmentally appropriate non-human primate model or pursue potentially new avenues for clinical research directly in pregnant human subjects. Third, our sample size for RNA-seq, though considered acceptable practice^65^, only captures big-fold changes in significant differential gene expression. Therefore, we cannot the exclude the possibility of a small effect of anesthesia and surgery on the cortical transcriptome, but we are reassured nonetheless because such effects would be similarly distributed across both treatment conditions. Fourth, even though our low-dose Oxt infusion for the first 4 h would have resulted in cervical ripening as demonstrated in laboring women^66^, we are unable to provide objective evidence to support that assumption. Nevertheless, because birth of the pups occurred predictably, it is unlikely to be a concern. Fifth, we did not compare the extent to which Oxt increases intrauterine pressure compared to saline. Because we had biological validation of pup birth, our objective was to capture the temporal relationship between the initiation of Oxt and the rise in intrauterine pressure rather than assess differences in intrauterine pressure between Oxt-induced and spontaneous labor that have been characterized previously^67^. Sixth, the absence of oxidative stress at birth does not exclude the possibility of an effect at a later time point. Finally, a thorough pharmacokinetic profiling of Oxt would have been ideal but was beyond the scope of our study. This limitation, however, is partly offset by the poor correlation between maternal plasma Oxt level and uterine contractility in laboring women^68^.

In conclusion, we provide a viable and realistic animal model for labor induction and augmentation with Oxt and demonstrate its utility in addressing clinically relevant questions in obstetric practice. Adoption of our model by other researchers would enable new lines of investigation related to the impact of perinatal Oxt exposure on the mother-infant dyad.

## Materials and methods

### Study design

All animal experiments and methods reported here were approved by the Institutional Animal Care and Use Committee at Washington University in St. Louis (#20170010) and conducted in accordance with the institutional and ARRIVE guidelines.

### Development of the pregnant rat model for labor induction and augmentation with Oxt

The system consists of a subcutaneously placed iPRECIO infrared-controlled microinfusion pump (SMP-200, Primetech Corporation) connected to the right internal jugular vein in an embryonic day (E)18 Sprague Dawley dam (Charles River Laboratories) (Fig. 2). Briefly, the dam was anesthetized with 2% isoflurane followed by subcutaneous implantation of the iPRECIO pump approximately 2–3 cm below the nape of the neck and creation of a tunnel to deliver the pump tubing next to the internal jugular vein, into which it was secured in place with ligatures. The reservoir of the iPRECIO pump was primed with sterile normal saline prior to implantation and was pre-programmed to deliver an infusion rate of 10 μl/h for 72 h to keep the tubing patent until E21. Two hours before completion of the saline infusion at 72 h, the reservoir was accessed subcutaneously under brief isoflurane anesthesia to aspirate the saline and was refilled with 900 μl of Oxt (Selleck Chemicals, 50 μg/mL in normal saline). This was followed by the pre-programmed infusion rate of 5 μl/h for 4 h, 10 μl/h for 4 h, 20 μl/h for 4 h, and 30 μl/h for 12 h (iPRECIO Management System) (Supplementary Fig. S2).

### Validation experiments

Though the witnessed birth of pups offered functional validation, we examined the effect of Oxt on the uterine myometrium with molecular biological assays, immunohistochemistry, and telemetric assessment of changes in uterine pressure.

i. *OxtR gene expression:* Briefly, approximately 0.5 cm × 1 cm rectangular piece of myometrial tissue was harvested from the anti-mesometrial aspect of the uterus after 8–12 h of exposure to either Oxt (100 mcg/mL concentration) or saline. Sample processing and OxtR qPCR was performed with a custom TaqMan® OxtR probe as described by us previously^69^.
ii. *Western blot for OxtR expression:* Membrane-associated proteins were isolated from approximately 100 mg of uterine myometrial tissue using Mem-PER Plus Membrane Protein Extraction Kit (catalog# 89842, ThermoFisher Scientific, Inc.) following manufacturer's instructions and subjected to immunoblotting with appropriate positive and negative controls (Cat#: LY400333, Origene Technologies, Inc). Approximately 10 μg of protein per lane was electrophoresed and transferred to membrane using Bolt western blot reagents (bolt 4–12% Bis Tris gel, catalog # NW04125; bolt sample reducing agent, catalog B0009; bolt LDS sample buffer, catalog # B0007; iBlot2 dry blotting system). The membrane was subsequently blocked with TBST buffer (catalog # S1012, EZ Bioresearch) containing 5% milk for 1 h at room temperature on a shaker. Following a brief wash with TBST buffer, the membrane was immunoblotted overnight at 4 °C on a shaker with primary rabbit anti-OxtR antibody (catalog # TA351476, OriGene Technologies), at a dilution of 1:250 in 5% milk-TBST buffer. HRP-conjugated secondary antibody (anti-rabbit IgG, catalog #7074, Cell Signaling Technology, Inc.) was used at a dilution of 1:1000 for 1 h at room temperature on a shaker. Immunoblots were incubated with ProSignal® Dura ECL Reagent (catalog #20-301, Prometheus Protein Biology Products) for 2 min at room temperature and detection of bound antibody was achieved with LI-COR Fc imaging system (LI-COR Biosciences Inc.) and the band concentrations were analyzed with Image Studio™ Ver. 5.2. For loading control, the membrane was stripped using Western ReProbe PLUS (catalog# 786-307, G-Biosciences, Inc) according to manufacturer’s instructions and immunoblotted with HRP-conjugated β-Actin (mouse monoclonal IgG), at a dilution of 1:1000 (catalog # sc-47778, Santa Cruz Biotechnology Inc.) for 1 h.
iii. *Immunohistochemistry:* To qualitatively confirm our western blot results, we performed immunohistochemistry for OxtR expression in the uterine myometrium. Briefly, 5-μm frozen sections of uterine myometrium embedded in OCT compound were obtained using Leica CM1510 S cryostat and immunostained for phosphorylated myosin light chain kinase (1:200 rabbit anti-mouse phosphomyosin light chain kinase, Invitrogen) and imaged with the Zeiss Axioskop 40 microscope. OxtR protein expression was assessed by immunostaining with goat anti-rat OXTR antibody (1:100; Origene) and revealed with Alexa Fluor® 594 labeled rabbit anti-goat antibody (1:300, Invitrogen). All primary antibodies were incubated overnight at 4ºC followed by a 1 h incubation with secondary antibodies at room temperature. Imaging was performed with Olympus BX60 fluorescence microscope with designated filter sets.
iv. *Uterine telemetry:* To assess whether initiation of Oxt was temporally associated with increase in intrauterine pressure, we performed pressure recordings with telemetry as described previously by us for mice^70,71^. Briefly, under isoflurane anesthesia and sterile precautions, we inserted a pressure catheter in the right horn between the uterine wall and the fetus under sterile precautions during pump implantation in E18 dams. To minimize the possibility that telemetry recordings could represent spontaneous labor, we advanced the time of replacement of saline with Oxt to 48 h instead of 72 h (i.e., E20—two days before term gestation). The pressure catheter was connected to a PhysioTel PA-C10 transmitter (Data Sciences International) placed in the lower portion of the abdominal cavity. Telemetry recordings were performed at 500 Hz with Dataquest ART data acquisition system version 4.10 (DSI) sampling every 5 min for 15 s intervals for 6 h at baseline, followed by recordings 48 h later when Oxt was initiated and continued until the birth of the pups.

### Effect of in utero exposure to anesthesia and surgery on the neonatal cortical transcriptome

To rule out the possibility of adverse effects on the fetal brain from intrauterine exposure to anesthesia and surgery during pump implantation^41,42^, we examined the cortical transcriptome of newborn pups delivered spontaneously by unhandled *vs*. surgically implanted dams. Briefly, 2 brains from spontaneously delivered newborn pups of either sex were collected within 2 h of birth from 6 dams (n = 3 each for spontaneous labor and saline-filled iPRECIO pump at E18). Total RNA was extracted from the right cerebral cortex using RNAeasy kit (Qiagen) and subjected to RNA-seq (Genome Technology Access Center core facility). Only RNA with RIN > 9.5 were used for RNA-seq. Processing of samples, sequencing, and analysis were done as described by us previously^69^ and detailed in the Supplementary Materials and Methods.

### Assessment of biomarkers of oxidative stress in the newborn brain

Briefly, brains were isolated from vaginally delivered newborn pups within 30 min to 2 h after birth, snap frozen, and stored at -80 °C for oxidative stress assays. Cortical lysates were prepared according to the assay type and protein concentration was determined using BCA Protein Assay Kit (ThermoFisher Scientific) prior to the assays. All assays were performed in duplicate, and fluorescence/absorbance was read with Tecan Infinite® M200 PRO multimode plate reader using appropriate filter sets as recommended by the manufacturer. We assayed for total free radicals (OxiSelect™ In Vitro ROS/RNS Assay Kit, #STA-347), 4-hydroxynonenal (lipid peroxidation marker, OxiSelect™ HNE Adduct Competitive ELISA Kit, # STA-838), protein carbonyl (marker of oxidative damage to proteins; OxiSelect™ Protein Carbonyl ELISA kit, # STA-310), total glutathione (OxiSelect™ Total Glutathione Assay kit, # STA-312), and total antioxidant capacity (OxiSelect™ TAC Assay Kit, # STA-360). All assays were purchased from Cell BioLabs, Inc (San Diego, CA) and performed as described by us in detail previously^72^.

### Expression of genes mediating oxidative stress in the newborn brain

From the same set of experiments as above, brains were isolated from additional pups born after exposure to either Oxt or saline (n = 6–8 per group), snap frozen, and immediately stored at -80 °C. Processing of total RNA for gene expression experiments was performed as described by us previously^69^. Expression levels of 16 genes relevant to oxidative stress (*Mtnd2*, *Mtnd5*, *Mtcyb*, *Mt-co1*, *Mt-atp8*) and antioxidant (*Sod1*, *Sod2*, *Gpx1*, *Gpx4*, *Prdx1*, *Cat*, *Gsr*, *Nox3*, *Nox4*, *Txnip*, *Txrnd2*) pathways were assayed in duplicate along with four endogenous housekeeping control genes (*18S rRNA*, *Gapdh*, *Pgk1*, and *Actb*) and reported as described previously^69^.

### Statistical analysis

Data outliers were detected and eliminated using ROUT (robust regression and outlier analysis) with Q set to 10%. Because our pilot experiments with a higher dose of Oxt (100 mcg/mL concentration) showed no sex differences in the expression of oxidative stress markers in the newborn brain, all subsequent analyses were performed regardless of sex of the offspring. RNA-seq data were analyzed as described by us previously^69^. Quantitative data were analyzed with Welch’s t-test with *p* ≤ 0.05 considered significant, while oxidative stress gene expression data were analyzed with unpaired student’s t-test followed by Bonferroni correction with an adjusted *p*-value ≤ 0.003 considered significant. All analyses, with the exception of RNA-seq data, were performed on Prism 8 for Mac OS X (Graphpad Software, Inc, La Jolla, CA) and expressed as mean ± S.E.M.

### Paper presentation information

This abstract was presented for Oral Presentation at the 50th Annual Meeting of the Society for Obstetric Anesthesia and Perinatology, Miami, FL, May 9–13, 2018.

## Supplementary Information

Supplementary Information 1.

Supplementary Information 2.

Supplementary Information 3.

Supplementary Video 1.

Supplementary Video 2.

## Data Availability

The equipment needed to establish the model are commercially available and non-proprietary. All data needed to evaluate the conclusions in the paper are present in the paper and/or the Supplementary Materials. The RNA-seq data used in this publication have been deposited in NCBI's Gene Expression Omnibus (GEO) and are accessible through the GEO Series accession number GSE161122 (https://www.ncbi.nlm.nih.gov/geo/query/acc.cgi?acc=GSE161122).
