# Publisher Correction: Labor induction with oxytocin in pregnant rats is not associated with oxidative stress in the fetal brain

**DOI:** 10.1038/s41598-022-08529-x

**Published:** 2022-03-14

**Authors:** Tusar Giri, Jia Jiang, Zhiqiang Xu, Ronald McCarthy, Carmen M. Halabi, Eric Tycksen, Alison G. Cahill, Sarah K. England, Arvind Palanisamy

**Affiliations:** 1 Department of Anesthesiology, Washington University School of Medicine, St. Louis, MO USA; 2 Department of Obstetrics and Gynecology, Washington University School of Medicine, St. Louis, MO 63110 USA; 3 Department of Pediatrics, Washington University School of Medicine, St. Louis, MO USA; 4 Department of Genetics, Washington University School of Medicine, St. Louis, MO USA; 5 Department of Women’s Health, Dell Medical School, The University of Texas at Austin, Austin, TX USA; 6 Present Address: Department of Anesthesiology, Beijing Chaoyang Hospital, Capital Medical University, Beijing, China; 7 Present Address: Department of Radiation Oncology, Washington University School of Medicine, St. Louis, MO 63110 USA

Correction to: *Scientific Reports* 10.1038/s41598-022-07236-x, published online 24 February 2022

The HTML version of the original version of this Article contained an error in the legend of Figure 4, where a portion of the text was incorrectly split and captured as an image credit.

The PDF version was unaffected.

The original Article has been corrected.

